# Analysis of the Self-Healing Capability of Thermoplastic Elastomer Capsules in a Polymeric Beam Structure Based on Strain Energy Release Behaviour during Crack Growth

**DOI:** 10.3390/polym15163384

**Published:** 2023-08-12

**Authors:** Mohammed Dukhi Almutairi, Feiyang He, Yousef Lafi Alshammari, Sultan Saleh Alnahdi, Muhammad Ali Khan

**Affiliations:** 1 School of Aerospace, Transport, and Manufacturing, Cranfield University, Cranfield MK43 0AL, UK; feiyang.he@cranfield.ac.uk (F.H.); yousef.alshammari@nbu.edu.sa (Y.L.A.); s.nahdi27@gmail.com (S.S.A.); 2 Centre for Life-Cycle Engineering and Management, Cranfield University, College Road, Cranfield MK43 0AL, UK; 3 Mechanical Engineering Department, Engineering College, Northern Border University, King Fahad Road, Arar 92341, Saudi Arabia

**Keywords:** 3D printing, TPE origami capsule, embedded structure, self-healing mechanism, double cantilever beam test

## Abstract

The objective of this study was to investigate the elastic and plastic responses of 3D-printed thermoplastic elastomer (TPE) beams under various bending loads. The study also aimed to develop a self-healing mechanism using origami TPE capsules embedded within an ABS structure. These cross-shaped capsules have the ability to be either folded or elastically deformed. When a crack occurs in the ABS structure, the strain is released, causing the TPE capsule to unfold along the crack direction, thereby enhancing the crack resistance of the ABS structure. The enhanced ability to resist cracks was confirmed through a delamination test on a double cantilever specimen subjected to quasi-static load conditions. Consistent test outcomes highlighted how the self-healing process influenced the development of structural cracks. These results indicate that the suggested self-healing mechanism has the potential to be a unique addition to current methods, which mostly rely on external healing agents.

## 1. Introduction

The field of 3D printing smart polymers is growing rapidly. With various advanced manufacturing techniques, it is now feasible to produce sophisticated and costly materials while minimizing waste. Man-made polymers with inherent self-healing abilities can enable the repair of damage like cracks or scratches, extending the lifespan of products. By integrating dynamic or reversible bonds into the polymer structure, it becomes possible to achieve a harmonious combination of healing capabilities and strong mechanical properties [1,2,3,4,5,6]. There is still a significant amount of work that needs to be accomplished to effectively utilize self-healing mechanisms in practical applications, given that most previous research has been limited to laboratory-scale studies of self-repairing structural damage. Most of the existing methods for self-healing rely on external factors, such as cracks caused by heat or chemical reactions, to initiate the healing process. However, these mechanisms are typically only effective for significant damage and cannot be implemented in real-time without external intervention. This limitation applies to self-healing mechanisms used in 3D-printed products as well. Ensuring the accuracy and reliability of critical applications is of utmost importance [7,8,9].

A self-healing mechanism is a desirable property in many engineering applications, as it can extend the lifetime of structures and reduce the need for maintenance. One approach to self-healing is to use materials that can repair damage by themselves. This can be achieved by incorporating healing agents into the material, which can diffuse to the site of a crack and fill it in [4]. Another approach to self-healing is to use structures that are able to self-repair without the need for external healing agents [8], achieved by designing structures with features that can close cracks or gaps. One such feature is a crack arrestor, a device that can stop a crack from propagating. The healing agent then undergoes a series of chemical reactions, triggered by factors such as heat, moisture, or catalysts, leading to the formation of interfacial bonds and restoring the structural integrity [10].

A different approach to developing intelligent 3D-printed items involves incorporating innovative origami capsules, in which the printed part contains multiple layers. In particular, these capsules can be used to establish an artificial hormone network, enhancing the safety and reliability of 3D-printed products [7,9,10]. Utilizing standard fused deposition modelling, these capsules can be embedded during the printing process, offering a cost-effective solution for large-scale production. This approach is akin to how the human hormone system functions when the body encounters a virus or bacteria. Through the use of origami-inspired capsules activated by strain removal, the capacity for self-healing within components or structures can be significantly revolutionized. Any type of surface or subsurface damage can trigger the initiation of strain removal in the entire component, with the capsules unfolding and expanding to facilitate strain removal at a subsurface level [11,12,13,14,15].

However, to achieve the opening or activation of these capsules when strain is released due to the initiation or growth of cracks within a structure, it is crucial to have a thorough understanding of their mechanical properties, especially when they are embedded. The introduction and control of this process involve establishing a functional connection between the initial stress applied to the embedded capsules, the movement of the folded parts in a direction that relieves the strain, and the amount of strain that is released.

Numerous research studies have investigated the self-healing capabilities of polymeric materials in beam structures. These studies have focused on characterizing the healing efficiency under various loading conditions, optimizing the capsule geometry and content, evaluating the effects of healing agents on mechanical properties, and exploring the long-term durability of self-healing mechanisms [16,17,18,19]. The results have demonstrated the potential of TPE capsules in reducing crack propagation and improving the structural performance of polymeric beam structures.

Another study investigated the self-healing capability of TPEs using a fracture mechanics approach [20,21,22,23]. Ponnusami et al. [24] proposed an analytical model for predicting the self-healing efficiency of a cracked beam structure with encapsulated healing agents, considering the strain energy release rate during crack growth and the healing efficiency of the encapsulated healing agents. Qian et al. [25] studied the self-healing behaviour of a cracked beam using shape memory alloy wires, analysing the strain energy release behaviour during crack growth and its impact on the shape recovery of the shape memory alloy wires. These articles underscore the significance of understanding the strain energy release behaviour in self-healing materials and structures and provide insights into various self-healing mechanisms, such as microcapsules, healing agents, and shape memory alloys.

Hernandez et al. developed a more efficient model for studying origami structures [26,27]. Li and You designed an origami beam that could better absorb energy than conventional beams [28]. However, their models were not perfect and needed further validation. Despite these limitations, origami-based encapsulation has shown promise [29], but more research is needed to test its mechanical strength and healing properties on stronger and stiffer materials [30,31,32,33,34,35].

Lee’s research [36,37] focused on studying the elastic energy characteristics of curved–creased origami using an elastic bending machine. Their objective was to examine the bending behaviour of the material. They developed an origami design model with different folds to create a 3D structure meeting a specific buckling criterion. The model was validated through experimental tests. Additionally, another study by Lee [36] showed that curved–creased laminated surfaces with a skewed shape could be used to construct compliant structures for energy absorption, though not specifically for self-healing purposes. Once the findings were verified, the researchers simulated a healing process using origami capsules.

In this work, we will focus on analysing the self-healing capability of thermoplastic elastomer (TPE) capsules in polymeric beam structures, specifically focusing on the strain energy release behaviour during crack growth. Understanding the strain energy release rate of a material enables us to design structures that are more resistant to crack growth. Tests were conducted to observe the effects of the material and binder on two types of 3D-printed beams. These tests were specifically designed to measure the elastic and plastic responses of the beams under various bending loads. Utilizing the results obtained from these tests, we developed a novel self-healing mechanism based on origami capsules triggered by crack propagation caused by strain energy release within a structure.

The main beam structure was enhanced by embedding cross-shaped origami capsules made of TPE. These capsules contained four small beams that had the ability to fold or deform elastically. When a crack developed in the main beam, the strain energy released caused the small beams to unfold in the direction of the crack, thereby increasing the resistance to crack propagation. The effectiveness of this self-healing mechanism was confirmed through delamination tests on a double cantilever specimen under quasi-static load conditions. The repeated tests demonstrated that the self-healing mechanism effectively improved the structural strength and resistance against crack growth. These results suggest that the proposed self-healing mechanism could be a valuable addition to current practices, which primarily rely on external healing agents.

## 2. Methodology

**Figure 1:**
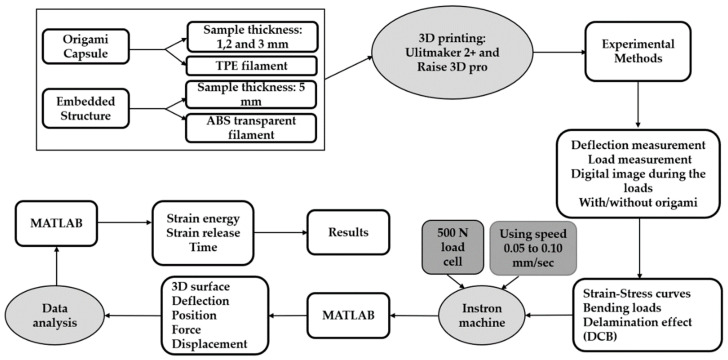
Methodology diagram.

This section provides an overview of the different methods employed for the preparation and characterization of the samples. Specifically, the application of bending loads on the polymeric origami capsule’s end was conducted to enhance our comprehension of its elastic and plastic properties. The research was divided into four main stages, as illustrated in Figure 1. The initial stage involved selecting the appropriate polymeric material, preparing the specimens, and implementing experimental methods. The second stage focused on designing the polymeric structure, including the TPE origami capsule. In the third phase, experiments were planned and performed to investigate the characteristics of the samples. These experiments involved conducting tests to measure the bending moment and delamination. In the fourth phase, a tensile testing machine was utilized to generate strain–stress curves, determine points of bending, and observe the impact of delamination under a single bending moment, as depicted in Figure 1.

### 2.1. Selection of Materials

The material chosen for this research was TPE. This is a flexible and rubber-like material that processes like plastic and exhibits good mechanical properties, good solvent and abrasion resistances, good shape retention, good weather resistance, low specific gravity, and high impact strength. Thermoplastic elastomers (TPEs) are a class of materials that combine the characteristics of both thermoplastics and elastomers. They offer a unique combination of flexibility and resilience, making them versatile and suitable for various applications. TPEs are used in a wide range of industries due to their excellent mechanical properties, ease of processing, and cost-effectiveness. TPEs can be processed using methods like injection moulding or extrusion, where they are heated for shaping. Once cooled, they regain their original elastic properties, making them recyclable (similar to thermoplastics).

TPE exhibits greater elasticity compared to ABS and is highly suitable for folding into capsule shapes. TPE has additional advantages over other polymers, such as being highly flexible, strong, and tactilely pleasant.

ABS transparent was selected as the embedded structure for its specific properties and characteristics. ABS transparent is a versatile material that offers transparency and desirable mechanical properties, making it a preferred choice in applications where both visibility and strength are required. Its processability and recyclability further enhance its attractiveness for various industries. This material was chosen due to its transparency, allowing for the visual inspection and observation of the self-healing process. The transparency of ABS enables the direct observation of any crack propagation or healing occurring within the structure. This feature is essential for evaluating the effectiveness of the self-healing mechanism and ensuring its reliability. By selecting ABS transparent as the embedded structure, we could effectively combine the benefits of transparency and self-healing capabilities in the designed system.

### 2.2. Specimen Preparation

**Figure 2:**
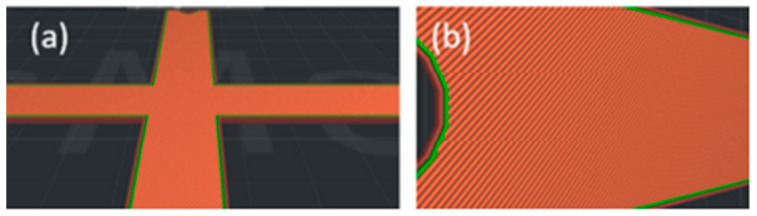
Printing directions of TPE: (**a**) 0 orientation; (**b**) ±45 orientation.

**Figure 3:**
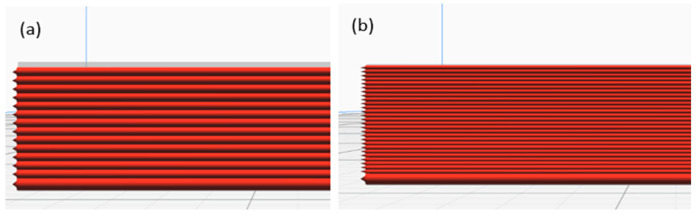
Layer thickness: (**a**) 0.10 mm and (**b**) 0.20 mm.

A Raise3D Pro printer was employed to create the origami capsule and embedded structure beam. During the 3D printing process of the capsule, two parameters, namely layer thickness and orientation, were adjusted. The printer platform was heated to 60 °C and the speed of printing was set at 20 mm/s. To ensure an adequate supply of material, a minimum of 500 g of filaments with a diameter of 1.75 ± 0.05 mm was used. The slicer program utilized this filament diameter to calculate the necessary feed rate for the printing process [38,39,40]. The specific mechanical printer parameters can be found in Table 1 and visual representations of the printer are shown in Figure 2 and Figure 3.

**Figure 4:**
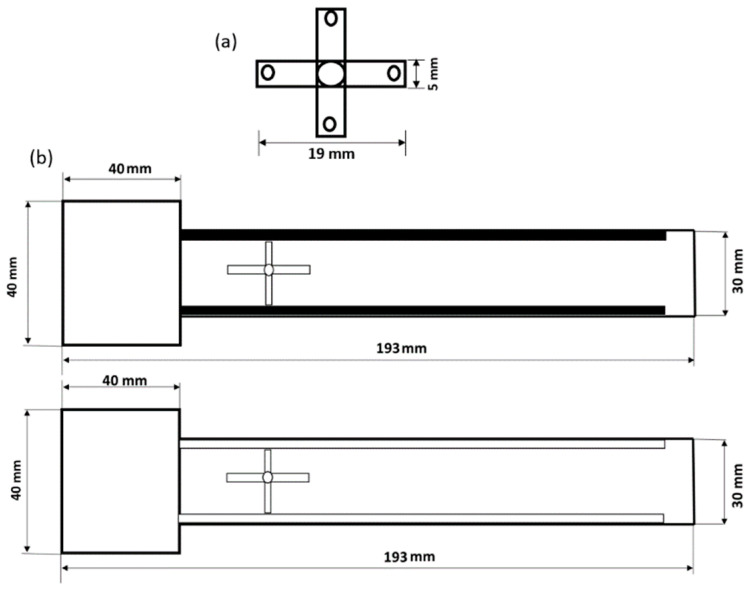
Specimen geometry: (**a**) origami capsule TPE; (**b**) double cantilever beam (DCB) (with hole and pillars).

The specimen was designed as an origami capsule and an embedded structure. Figure 4 illustrates the design of the ABS-embedded structure, including its dimensions. The origami capsule was created using Inventor software 2020, as depicted in Figure 4a, with thicknesses of 1, 2, and 3.0 mm, length of 19 mm, and width of 5 mm. Figure 4b displays the dimensions of the specimen, which maintained a consistent length of 193.0 mm, width of 30 mm, and thickness of 5 mm throughout all the conducted tests.

**Figure 5:**
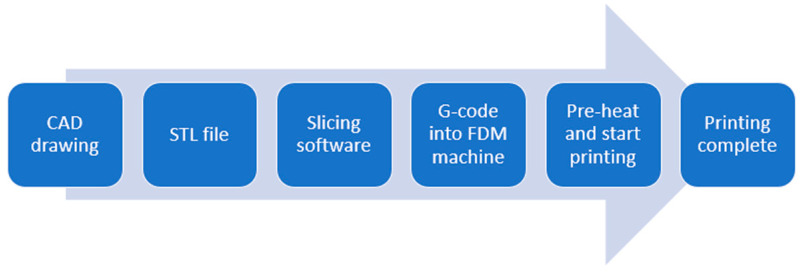
The process of 3D printing involves several steps, starting from the initial drawing to the final fused deposition.

The G-code files needed to print the specified specimens on a Raise3D pro2 printer were generated using Idea Maker software 3.6.1. Figure 5 illustrates the entire 3D printing process, including the initial design and the fused deposition.

For each capsule and embedded structure, a minimum of three samples were produced using 3D printing. The printing process began with the printing of TPE (thermoplastic elastomer) samples, followed by blends consisting of 5%, 10%, and 20% TPE by weight. The nozzle temperature was consistently set to 60 °C for all the capsules. A printing speed of 40 mm/s was maintained, and the print bed temperature was set to 60 °C. Similarly, ABS (acrylonitrile butadiene styrene)-embedded structures were also printed using blends containing 40%, 60%, and 80% ABS by weight. The printing parameters for ABS were a constant printing speed of 60 mm/s, a nozzle diameter of 0.4 mm, and print bed temperatures of 80 °C and 100 °C [8].

### 2.3. Experimental Design

In this study, bending strain and delamination tests were performed on both the origami capsule and origami capsules integrated into structures. To conduct these experiments, an Instron 5944 universal testing machine (UTM) was utilized.

#### 2.3.1. Origami Capsule

Using a design-of-experiment methodology, the parameters for this study were set as shown in Table 2. A TPE origami capsule was produced. The sensor was calibrated three times for each of the following capsule thicknesses: 1.0 mm, 2.0 mm, and 3.0 mm. The applied load and deflection data were measured and recorded, and the data were plotted using Excel.

#### 2.3.2. Origami Capsule Embedded Inside the Structure of the Beam

A straightforward experiment was conducted to measure the load and displacement. This was achieved by performing a delamination test on specimen beams made from ABS polymer, as indicated in Table 3. Subsequently, the stress/strain relationship was assessed both with and without the presence of the origami capsule.

First, it was proposed that specimens with origami structures would exhibit greater tensile strength and resilience compared to specimens without origami structures. To test this hypothesis, two 3D-printed hollow beam prototypes were created, one with an adhesive-encapsulated origami structure and one without. Force versus displacement profiles were obtained to analyse the stress–strain relationship. The F/D curves were converted into stress/strain curves by dividing the force values by the cross-sectional area of the beam and the displacement values by the initial gauge length. Table 4 presents a diagram illustrating the experimental procedure utilized.

### 2.4. Experiment Procedure and Setup

**Figure 6:**
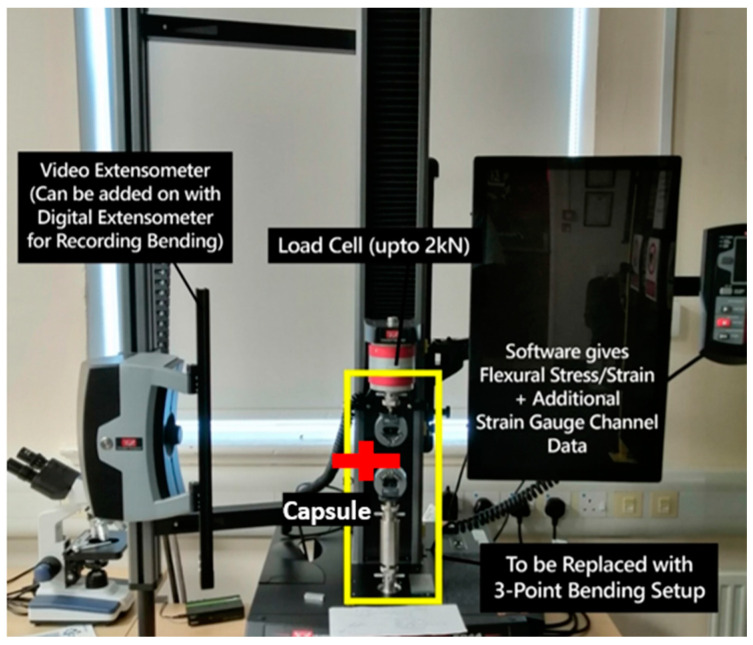
Deflection points of the end load of the origami capsule.

In this experiment, a micrometer was used to measure the deflection at the end of the origami capsule, as depicted in Figure 6. Before conducting the experiment, the dimensions of the origami capsule were assessed using Inventor software 2020, and the specific capsule dimensions can be found in Table 2 above.

The primary objective of the experiment was to assess how the geometry and internal density of materials impact their elastic properties. Furthermore, this study aimed to characterize the unique properties crucial for the design and production of customized products using additive manufacturing (AM) techniques. To examine the elasticity of each type of specimen concerning the interior lightening percentage and evaluate the self-healing mechanism, a specific capsule shape (star shape) was designed. These experiments were performed to measure the deflection at the end of the capsule (see Figure 6). As mentioned before, the origami capsules underwent a total of 3 tests. The bending load tests were conducted on the UTM machine, with a maximum applicable applied load of 106 g and a maximum deflection of 19 mm. All tests were recorded on camera to facilitate a comparison of the capsules’ performances.

It is essential to note that the primary goal of this research was to observe the capsules’ behaviour and their effectiveness in healing cracks before they propagate. Consequently, the bending tests were carried out under applied loads until the deflection point of the origami capsule was reached.

#### 2.4.1. Polymeric Origami Capsule Behaviour

Instead of using a basic ABS beam, a modified beam with an origami insert in a “cross” configuration was utilized, as outlined in Table 2. The origami capsule was subjected to various loads, and measurements were taken using a camera, signal conditioning unit, and data logger. The purpose of subjecting the TPE to a bending load was to gain insight into its elastic and plastic characteristics. The stresses were determined based on the applied load from the universal testing machine (UTM). The deflection of the capsule was determined by analysing video images captured during the loading process, which provided an overall understanding of the beam’s response to the bending load.

**Figure 7:**
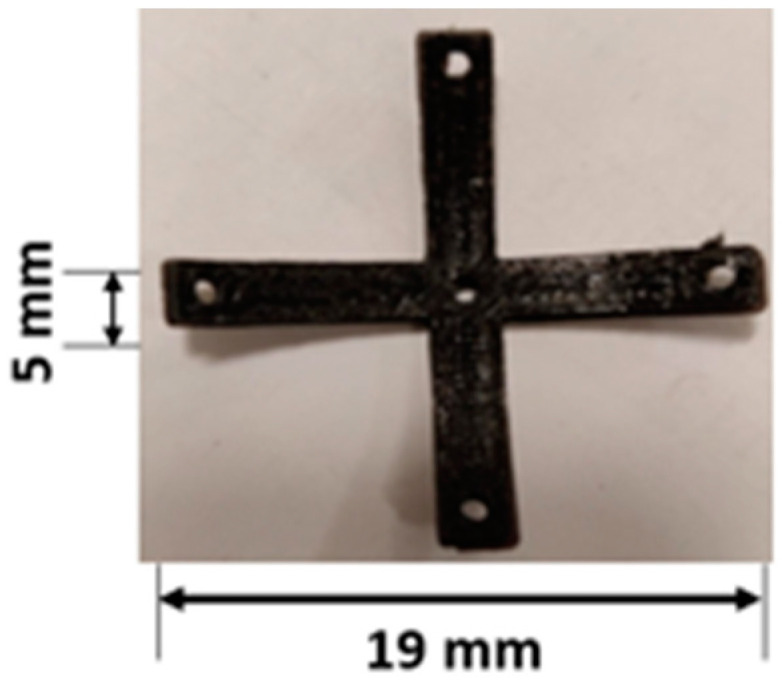
TPE origami capsule “cross”.

The experiment involved three thicknesses of 1.0, 2.0, and 3.0 mm. For each trial, a camera was positioned to measure a specific value, and consistent deflection values were captured. The experiment was repeated three times, and the average of the results was calculated and recorded. To assess the feasibility of various capsules, origami capsules with different shapes were designed using Inventor software 2020, as illustrated in Figure 7. The capsule designs were created in a way that their geometric characteristics remained within the initial pre-stressed conditions.

#### 2.4.2. Analysis of the Beam’s Behaviour when the Origami Capsule Is Present within it

**Figure 8:**
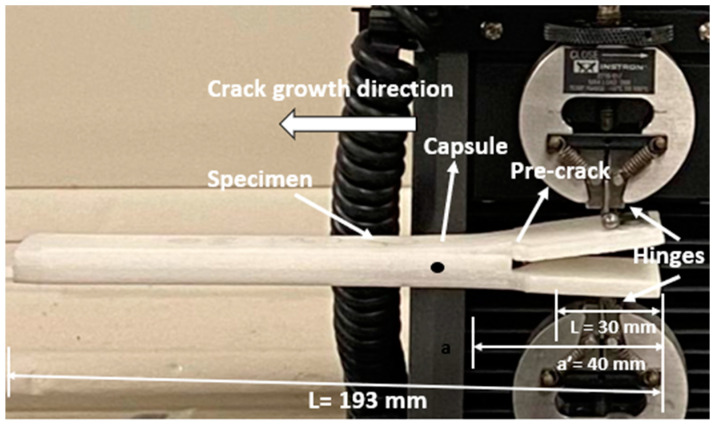
The experimental setup and demonstration of pre-cracking in the double cantilever beam (DCB).

The experimental design and setup for the delamination test are depicted in Figure 8. The structures used in the test were identical to those shown in Figure 4b, measuring 30 mm in width and 193.0 mm in length. These beams had a pre-crack of 40 mm running longitudinally from the front, as shown in Figure 8. To facilitate the testing process, 30 mm wide end tabs were attached to the external sides of the samples on both sides of the pre-crack. The tabs were firmly attached to an electromechanical uniaxial testing machine that had a 500 N load cell. The double cantilever beam (DCB) tests were carried out on an ITM, which provided adjustable displacement rates between 0.05 and 0.10 mm/s. Each quasi-static DCB measurement was repeated three times using the universal testing machine (UTM). The load applied was measured using a load cell connected to the tensile testing machine. Additionally, a camera was used to measure the opening displacement and the length of the fissure.

Since the ABS transparent beams were used in the study, crack length measurements were directly taken from the side using a camera during the delamination test. The origami capsule was utilized to determine the energy release rates and critical energy release rates. To prevent any permanent deformations that could indicate undesired energy dissipation, a non-monotonic programmed loading history was implemented. This involved conducting eight cycles of loading and unloading for each sample, all at the same displacement rate. The machine was programmed to define the maximum displacement during loading for each cycle, while a minimum force of 5 N was set during unloading to prevent the compression of the test specimen. This approach aimed to verify the absence of any unwanted sources of energy dissipation.

## 3. Results and Discussion

### 3.1. The Bending Moment of TPE “Cross” Origami Capsule Test Results

**Figure 9:**
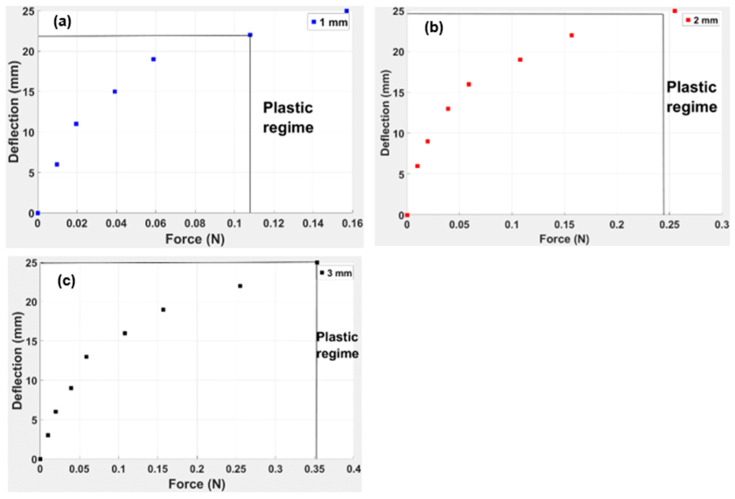
Deflection of end-loaded simple TPE capsule at various thicknesses: (**a**) 1 mm, (**b**) 2 mm, and (**c**) 3 mm.

The TPE material beam displacement response was tested for different thicknesses at variable loads applied at the free end of the capsule, as indicated in Figure 9. Theoretically, the deflection of the TPE “cross” origami capsule is directly comparable to the applied force and inversely proportional to the capsule thickness, which is evident in Figure 9. The highest deflection is observed for the capsule at more increased thickness, whereas the shaft for lower thickness tends to show an elastic region almost the half magnitude of the force. It is also evident that the maximum pressure at a 1 mm beam thickness is 0.15 N and the deflection is 21 mm; at a 2 mm beam thickness, these are almost 0.23 N and 24 mm; and at 3 mm thickness, the deflection is 25 mm at 0.35 N force. The elastic region and plastic region of the beam consolidate at higher beam thicknesses, based on Equation (1).

(1) $$\mathrm{Deflection}\;\propto \frac{f(force)}{f(thickness)}$$

The force and deflection data plotted on a graph can be used to determine the amount of strain energy in the beam. This analysis confirms that all of the applied stress on the beam is transformed into strain energy, as shown in Equation (1).

(2) $$\mathrm{Maximum}\;\mathrm{strain}\;\mathrm{energy}\;\mathrm{of}\;\mathrm{beam}\;(U)=\frac{\sigma^{2}}{2E}\;Ba$$

In this equation, the variable 𝜎 represents the applied stress, *E* represents the elastic modulus of the material, 𝐵 represents the thickness of the capsule, and *ɑ* represents the length of the capsule.

$$\mathrm{Maximum}\;\mathrm{strain}\;\mathrm{energy}\;\mathrm{of}\;\mathrm{the}\;\mathrm{beam}\;\mathrm{at}\;1\;mm\mathrm{capsule}\mathrm{thickness}\;=\frac{4.709^{2}}{2\;\times \;14}\;\times \;5\;\times \;25=98.986\;Nmm$$

$$\mathrm{Maximum}\;\mathrm{strain}\;\mathrm{energy}\;\mathrm{of}\;\mathrm{the}\;\mathrm{beam}\;\mathrm{at}\;2\;mm\mathrm{capsule}\mathrm{thickness}\;=\frac{1.912^{2}}{2\;\times \;14}\;\times \;10\;\times \;25=32.674\;Nmm$$

$$\mathrm{Maximum}\;\mathrm{strain}\;\mathrm{energy}\;\mathrm{of}\;\mathrm{the}\;\mathrm{beam}\;\mathrm{at}\;3\;mm\mathrm{capsule}\mathrm{thickness}\;=\frac{1.177^{2}}{2\;x\;14}\;\times \;15\;\times \;25=18.560\;Nmm$$

The calculated values show that the strain energy for 1 mm capsule thickness is the highest, almost 98.986 Nmm; for 2 mm capsule thickness, it is 32.674 Nmm; and it is the least for 3 mm capsule thickness, which is 18.560 Nmm. This shows that higher values of capsule thickness produce the minimum amount of strain energy, and lower values of beam thickness produce the maximum amount of strain energy.

The vertical lines in the force and deflection graph show the initiation of the plastic regime and the end of the elastic region. At higher thickness, the beam could sustain a high force magnitude and show maximum deflection before entering the plastic region. However, for the minimum thickness of the 1 mm beam, the elastic region only sustained a 0.11 N load (see Figure 9). This is because the TPE “cross” is already a flexible rubber-like material; at a minimum beam thickness, the overall load-bearing capacity of the beam reduces. Hence the beam can withstand minimum deflection before entering the plastic region.

**Figure 10:**
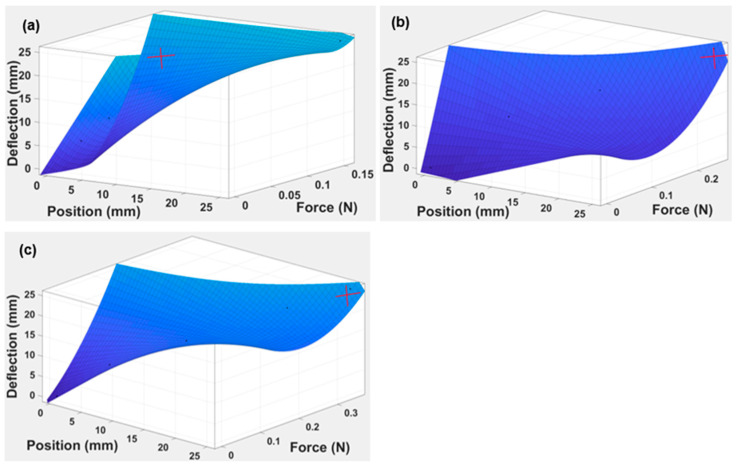
The 3D gradient graph illustrates the correlation between applied load (N), position (mm), and deflection (mm) for three different thicknesses of the TPE “cross” capsule. These thicknesses were (**a**) 1 mm, (**b**) 2 mm, and (**c**) 3 mm.

With the obtained data for the maximum deflection applied at different loads, there is a further need to assess different parameters affecting the beam. A 3D graph was plotted to correlate the behaviour of the beam with the action of deflection, force, and position, as indicated in Figure 10.

The estimation is performed using a 3D surface graph, as indicated in Figure 10. The three-variable graph shows the parametric relationship between the load, position, and deflection of the capsules. The changes in the maximum deflection of the beam were calculated for different values of loads. It is confirmed from the gradient that as the thickness of the capsule increases from 1 to 3 mm, the beam tends to deflect at its maximum position.

For the 3D graph surfaces, the approximate polynomial Equation is given as:

(3) $$\mathrm{Deflection}\;\mathrm{of}\;\mathrm{the}\;\mathrm{beam}\;(f=\mathrm{force},\;y=\mathrm{position}\;\mathrm{at}\;\mathrm{any}\;\mathrm{point})=T_{00}+T_{10}f+T_{01}y+T_{20}\;f^{2}+T_{11}\;fy$$

where, $T_{00},T_{01},T_{10},T_{20}$, and $T_{11}$ are the coefficients of the polynomial.

The results of the coefficients at various TPE capsule thicknesses are shown in Table 5.

The polynomial equation for determining the thickness of each capsule can be easily obtained by inserting the coefficients into Equation (3).

**Figure 11:**
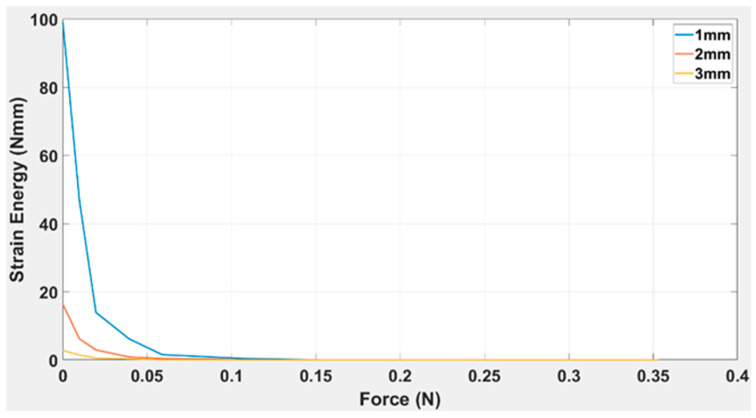
Strain energy vs. applied load for a simple TPE “cross” origami capsule of variable thicknesses (1 mm, 2 mm, and 3 mm).

The curves, as indicated in Figure 11, are plotted for strain energy (Nmm) as a function of force (N) applied on a single TPE “cross” origami capsule for (a) 1 mm, (b) 2 mm, and (c) 3 mm thickness. The legend in the top right corner indicates that the maximum strain value for a 1 mm capsule thickness was 98.98 Nmm; for 2 mm thickness, it was 16.33 Nmm; and for 3 mm thickness, it was 2.749 Nmm, respectively. This shows that the higher the capsule thickness value, the higher the strain energy and decay will be over time. Notably, at a higher value of beam thickness it takes a longer time for strain energy to decay with the action of force, as the 1 mm beam thickness had the highest value of strain energy, and it showed a reaction up to 0.07 N force. Meanwhile, the 2 mm and 3 mm capsules had already decayed at 0.04 N force. The governing equation for the trendlines for various thicknesses is given below.

(4) $$f\left(t\right)=ae^{bt}$$

$$1.0\;mm\;\;\;\;\;\;f\left(t\right)=196.5e^{-70.73t}$$

$$2.0\;mm\;\;\;\;\;f\left(t\right)=35.68e^{-81.59t}$$

$$3.0\;mm\;\;\;\;f\left(t\right)=4.77e^{-57.75t}$$

**Figure 12:**
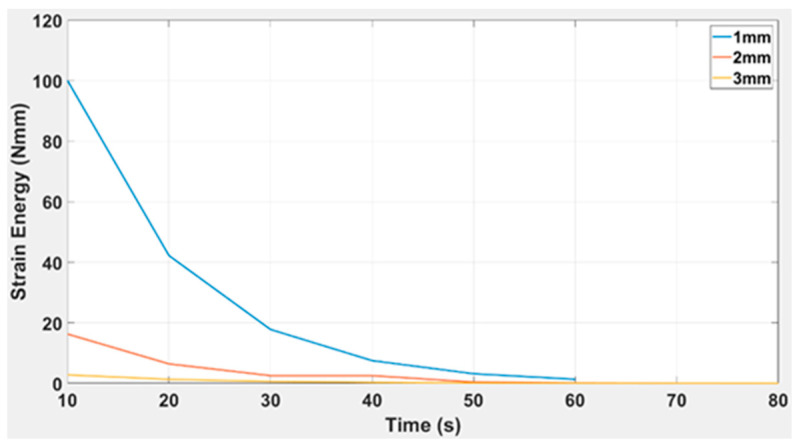
Strain energy vs. time for a simple TPE capsule of variable thicknesses (1 mm, 2 mm, and 3 mm).

The strain energy over time for different capsule thicknesses (1 mm, 2 mm, and 3 mm) is shown in Figure 12. The graph clearly demonstrates that the strain energy is significantly greater for the 1 mm thickness compared to the 3 mm thickness. However, the decay rate of strain energy is significantly lower for the 1 mm as compared to the 3 mm TPE capsule thickness. The governing equation for the trendlines for various thicknesses is given below.

(5) $$f\left(t\right)=ae^{bt}$$

$$\;1.0\;mm\;\;\;\;\;\;\;f\left(t\right)=237.4e^{-0.08637t}$$

$$2.0\;mm\;\;\;\;\;\;f\left(t\right)=41.33e^{-0.09308t}$$

$$3.0\;mm\;\;\;\;\;\;f\left(t\right)=5.926e^{-0.07551t}$$

According to the information provided in Table 5, it is stated that as the thickness value increases, the coefficient value also increases in absolute terms. This is why the 1mm beam thickness has the most negligible value of coefficients and the highest value of R-squared (0.9991), and vice versa. To simplify the variables, a curve has been plotted between the coefficients and beam thickness.

### 3.2. Discussion of the TPE “Cross” Origami Capsule Behaviour

This study is based on analysing the self-healing properties of the TPE material. The objective was to present an origami-based star design made of TPE material, which is further embedded into the beam to assess self-healing behaviour. From the material point of view, it is shown that the strain rate will increase the modulus of TPE as the material is highly compressible. It is also evident from Figure 9 that the plastic region for the 1 mm beam thickness occurs at the median position. The plastic region starts at the end for the 2 mm and 3 mm thicknesses. Although studies on the moment of inertia and the elastic modulus were not conducted, from the strain energy, it can be asserted that both parameters depend on dimensions and individual material properties.

TPE is characterised using the study’s extended research work and scope. The analytical process was similar to that of ABS and TPU [41,42], which involves the capsule deflection at different thicknesses, which is 1–3 mm in our case. From the analysis of 3D curves, as indicated in Figure 10, it is evident that the plastic regime achieves the maximum value of the applied load at 2 mm and 3 mm. The R-squared value for each thickness was 0.9991 for 1 mm TPE, 0.994 for 2 mm TPE, and 0.9965 for 3 mm TPE, as presented in Table 5. After plotting the strain energy vs. force in Figure 11 for all three capsules, it is proved that the strain energy is maximum at a higher capsule thickness value and decays gradually compared to the thinner capsules. The R-squared values are 0.9973, 0.9822, and 0.9956 for the 1 mm, 2 mm, and 3 mm thicknesses, which further supports the model’s validity. The graphical representation of strain energy over the period shows that the decay rate of strain energy is significantly lower at a higher beam thickness than at a smaller beam thickness (see Figure 12). Here, the model has R-squared values of 0.9944, 0.9989, and 0.9958 for the 1 mm, 2 mm, and 3 mm thicknesses.

To simplify the correlation results, the variable coefficients obtained from the model equations *T*_00_, *T*_01_, *T*_10_, *T*_11_, and *T*_20_ were graphed against the thickness values, as indicated in Table 5. The R-squared values for these graphs were 1.0, indicating an accurate fit of the data points in the model equations. Furthermore, from the experimental results, it is observed that the modulus value of the TPE “cross” origami capsule increases with the strain rate. The experimental results correlate with the material properties that define TPE as a flexible material with more flexible filaments than ABS and TPU [42]. The TPE is less hard than ABS and TPU, due to which the beam surface had a more rubbery texture [43]. This is why the maximum strain energy of TPE is only 100 Nmm, whereas, for TPU and ABS, it was 180 Nmm and 8000 Nmm, respectively. Therefore, the TPE polymer can be utilized in origami capsules for self-healing a low-rigidity structure.

### 3.3. Embedded Structure of the TPE “Cross” Origami Capsule Inside DCB Results

**Figure 13:**
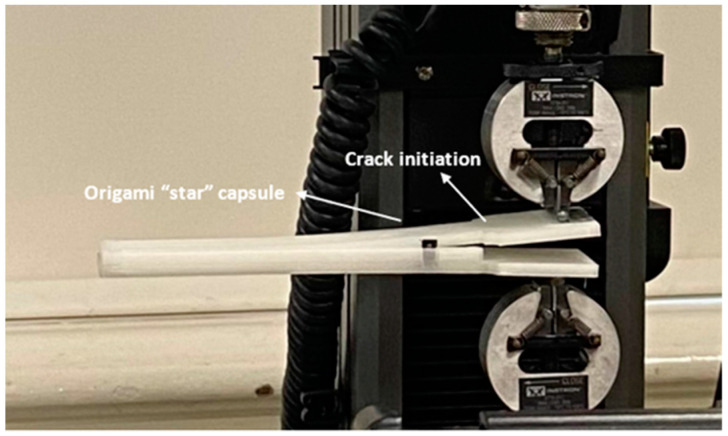
Assessing DCB crack length and displacement throughout the test.

**Figure 14:**
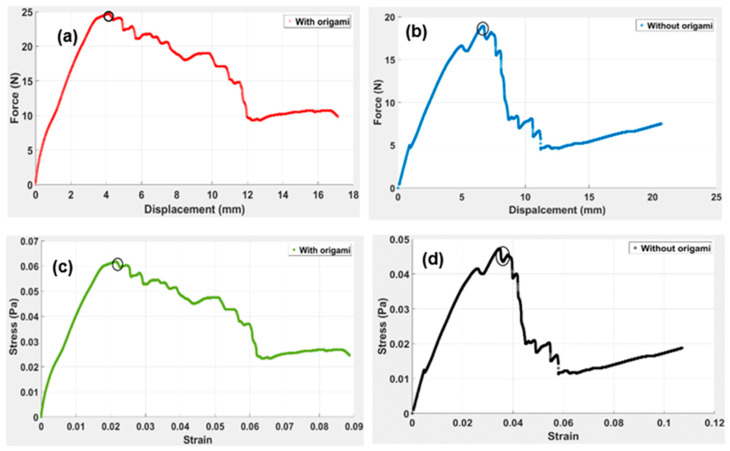
The length of cracks in the double cantilever beam (DCB) was measured under quasi-static conditions. The load-displacement plots for both beams with origami capsules and those without capsule are shown in (**a**–**d**).

The DCB model’s response allows us to assess the behaviour of the origami capsule and determine if it triggers a self-healing mechanism. The analysis involves evaluating the amount of stress released when the beam bends under the force applied. For the DCB experiment, instead of having interlaminar crack growth in the DCB, one end of the beam was trimmed down, as indicated in Figure 13. An HD camera was used to record the video of the experimental process. The entire setup involves taking a picture after every 10 s, which is used to evaluate the position of the crack from the tip to the point where the star origami is placed. From the results, it can be ascertained that the applied load on the beam is dependent on the strain energy release. The response of the applied force vs. displacement for the TPE-embedded DCB is shown in Figure 14.

The load–displacement graphs obtained from the beam embedded with the star origami structure are shown in Figure 14. The observation shows that there are some non-linearities in the load–displacement for the specimen. As indicated in Figure 14a–d, the graphical relationships are non-linear. In Figure 14a, the maximum amount of force is 25 N for the specimen with origami. In Figure 14b, the maximum resisted force for a specimen without origami is 17 N with a displacement of 22 mm. Noticeably, there is a substantial drop in force magnitude in both cases—with and without origami capsules. For the specimen with the star origami, the force dropped from 25 to 9 N. By contrast, the drop without origami was 16 to 6 N. Theoretically, the sudden failure is the highest to the lowest magnitude of the force that causes a jerk in the beam, which can lead to significant damage and even collapse of the DCB. Since the DCB with origami has a gradual decline in the force compared to without origami, it is evident that the inclusion of a star-shaped origami in the DCB can help reduce the sudden jerk due to force, which can reduce the chances of structural damage.

Utilizing parameters such as time taken by the beam and strain released, research can calculate the percentage error deviation between the DCB with origami and without origami using the formula:

(6) $$\mathrm{Strain}\;\mathrm{energy}\;\mathrm{release}\;\mathrm{due}\;\mathrm{to}\;\mathrm{crack}\;(U)=\frac{\sigma^{2}}{2E}\;B\pi a^{2}$$

The stress applied is represented by *σ* (N/m^2^), the elastic modulus of the materials is represented by *E* (N/m^2^), the area length is represented by *B* (mm), the area from the middle DCB to the open area is represented by π, and the length of the beam is represented by *ɑ* (mm).

Table 6 shows the experimental and theoretical values for the strain release during the testing of the beam, both with and without the star origami. The results show that the difference for origami is 0.183 and 0.233, which amounts to 18.31% and 23.3%, respectively. From these values, it is clear that the strain release without origami is greater than with origami. Therefore, the response of TPE in origami can help dampen the appreciable amount of strain release, preventing the beam from collapsing or being damaged.

**Figure 15:**
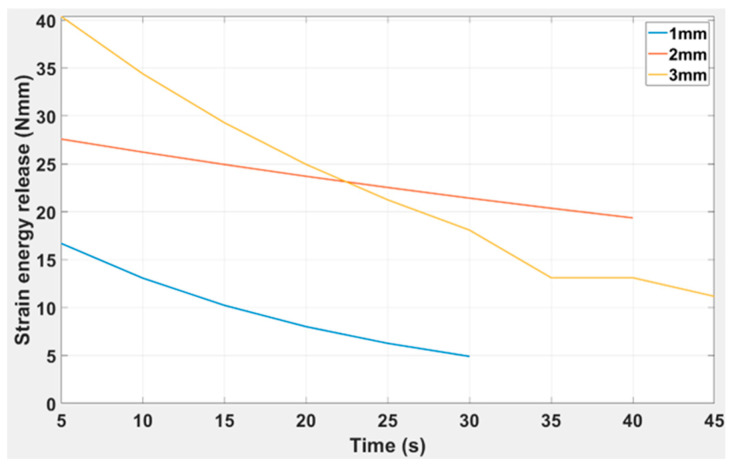
Strain energy release versus time for DCB: 1 mm, 2 mm, and 3 mm.

Figure 15 indicates the maximum amount of strain energy release for DCB of different thicknesses over a period of time, as shown in Equation (6). The maximum strain energy release was 18.2 Nmm, 28.7 Nmm, and 40 Nmm for the 1 mm, 2 mm, and 3 mm thicknesses, respectively. Similarly, the time at which the maximum strain release occurred was 30 s, 40 s, and 45 s, respectively.

The governing equation for the trendlines for various thicknesses is given below: it is clearly indicated that as the beam thickness increases, the maximum strain release time is reduced. The capability to retain maximum strain value also increases.

(7) $$f\left(x\right)=v_{1}x^{4}+v_{2}x^{3}+v_{3}+v_{4}x+v_{5}$$

$$1.0\;mm\;f\left(x\right)=-3.59\;\times \;10^{-8}x^{4}+3.78\;\times \;10^{-6}x^{3}-0.00013x^{2}+0.0018x-0.0032$$

$$2.0\;mm\;f\left(x\right)=-6.259\;\times \;10^{-5x^{4}}+0.0065x^{3}-0.248x^{2}+4.306x-7.94$$

$$3.0\;mm\;f\left(x\right)=-0.00054x^{3}+0.0023x^{2}+1.431x-1.658$$

**Figure 16:**
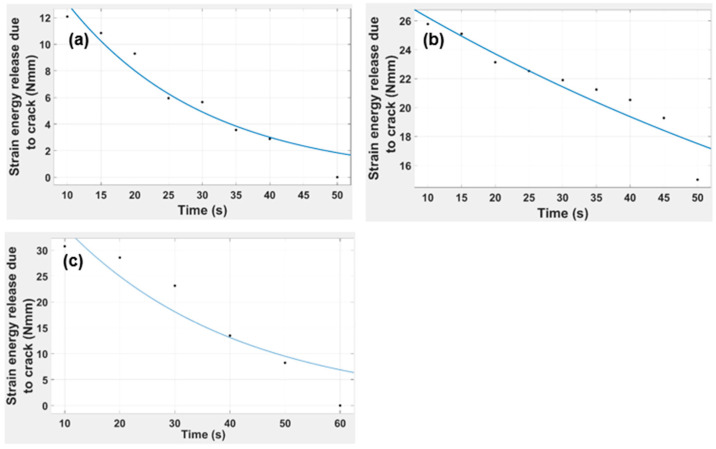
Graphical representation of DCB strain energy release due to a crack vs. time for DCB: (**a**) 1 mm, (**b**) 2 mm, and (**c**) 3 mm.

Figure 16 indicates the strain energy release due to the crack propagation versus time for the 1 mm, 2 mm, and 3 mm beam thicknesses. It is clear from the graphical trends that the value of strain release due to a crack increases as the thickness increases, and even the time to reach the zero value of strain release was extended for the higher thicknesses. The governing equation for the trendlines for various thicknesses is given below.

(8) $$f\left(t\right)=ae^{bt}$$

$$1.0\;mm\;f\left(t\right)=21.33e^{-0.04892t}$$

$$2.0\;mm\;f\left(t\right)=29.02e^{-0.01011t}$$

$$3.0\;mm\;f\left(t\right)=47.43e^{-0.03212t}$$

#### Discussion

This study analysed the behaviour of a star-shaped origami embedded in a DCB structure using TPE material. The method of testing the DCB is based on an Instron testing machine with displacement rates from 0.05 to 0.10 mm/s. The configuration from the energy release rates was also calculated using 15 printing parameters for different specimens. The results indicate that the TPE cross-origami is more reliable and robust in withstanding greater fluctuations than the beam without cross-origami. In addition, conducting a mathematical analysis that examines the relationship between force and displacement, as well as stress–strain curves, can be used to evaluate the validity of the hypothesis and research design.

Based on the experimental results, two sets of graphs were obtained for the two configurations, one with origami and one without origami (Figure 14a–d). With the origami capsule (see Figure 14a), the graph shows a nearly straight line from 0 N to the maximum force of 25 N, resulting in a total displacement of 4.8 mm. This indicates that the beam reaches its elastic limit and resistance at the maximum load. After this point, the DCB continues to bend, leading to an increase in displacement from the origin, reaching a maximum displacement of 17.5 mm, which represents the plastic region of the beam. This behaviour suggests that the origami capsule effectively absorbs and redistributes the applied force, allowing the DCB to bend and release strain without compromising the structural integrity. Such behaviour aligns with the concept of a self-healing mechanism, where the origami component absorbs and dissipates the energy associated with the force, mitigating damage to the beam. On the other hand, for the specimen without origami (Figure 14b), it is evident that the maximum force is resisted at 16 N with a total displacement of 6.5 mm. Notably, there is a significant drop in force from 16 N to 6 N, and after this failure, the beam collapses. Therefore, it is evident that DCB with origami withstands a greater amount of force upon maximum force, gradually releasing strain without damaging the beam. Experimental results theoretically support the previous [44] results, where the research work critically analysed the load vs. displacement test on the specimen. There was a clear difference in the lamination length for the two specimens with the sudden drop in load without origami.

These results indicate that the beam with the origami capsule has a better resistance against failure than the beam without the capsule. It has also been observed that the dimensions of the beam play an important role in defining the material’s strength. As per the study [43], different material observes different resistance toward multi-directional layups from crack propagation. The delamination resistance observed from the DCB test depends on the fibre orientation of the TPE. It is also indicated in Figure 14c,d that the capsule presented the maximum stress with origami at 0.06 Pa, and without origami it was 0.048 Pa. Upon comparing the load versus displacement curve, it became evident that the load increased linearly with the displacement. However, when the stitches broke, crack initiation led to a sudden drop in load. Furthermore, the results exhibited steady load fluctuation, similar to origami, indicating the material’s high tensile strength capable of bearing increasing loads even during crack formation.

In Figure 15, it is evident that as the beam thickness increases, the time at which the maximum strain release occurred was reduced, and the capability to retain maximum strain value also increases. The R-squared values are 0.927, 0.946, and 0.9041 for the 1 mm, 2 mm, and 3 mm thicknesses, which further supports the model’s validity. Notably, upon further study of the effect of beam thickness, it is shown in Figure 16 that the thickness of the beam and the strain energy release due to a crack are directly proportional to each other.

The stress–strain relationship of TPE in high-density stitched beams is discussed in [44]. Upon comparing the results from that research paper and our experimental results, it is evident that the load increases linearly with the displacement. Still, as the maximum force was reached, the stitches were broken, and the crack was initiated, causing a sudden load drop. The obtained results are similar to those in [45], which tested the crack propagation in a DCB made from polymeric composite materials [45]. The load fluctuations are also steady, indicating a high tensile strength to bear the increased load during the crack propagation.

## 4. Conclusions

The objective of the study was to analyse the response of self-healing beams made of TPE material when subjected to both elastic and plastic loads. In conclusion, the proposed TPE “star” origami capsule tends to absorb more fluctuation of load and displacement; however, upon comparing it with TPU and ABS, the results of TPE are ideal for less rigid material. The results for TPU and ABS are ideal for high-stress applications, but TPE is ideal for structures where even a small stress can lead to structural damage. Therefore, the hypothesis is that specimens made of TPE origami can propagate the self-healing properties when the load is axially supported. On the other hand, DCB structures tested without origami show less resilience and have low material tensile strength. Furthermore, the self-healing mechanism of the TPE origami capsule has been demonstrated and reported for the first time. These materials achieved a good balance of mechanical strength and self-healing ability. The self-healing mechanism proposed for these materials can contribute to the design of self-healing mechanisms in the future. TPE was confirmed to be capable of self-healing, and this should be developed for practical engineering applications. However, the next step in the research is to develop the relationship between the self-healing mechanism and a numerical model.

## Figures and Tables

**Table 1:** Printing parameters.

Parameters	Value
Nozzle size (mm)	0.4
Layer thickness (mm)	0.1, 0.2
Build orientation	0°, ±45°
Infill density (%)	100

**Table 2:** Designs of the origami capsules.

Origami Capsule Shape (TPE)	Thickness (mm)	Dimensions (mm)	Loads (g)
Cross	1.0, 2.0, 3.0	19 L/5 W	1, 2, 4, 6, 11, 16, 26, 36, 56, 86, 106

**Table 3:** Experiment scheme for the embedded structure.

Type of Specimen	Crack Length	Capsules	Mechanical Testing
With origami	40 mm	1 mm, 2 mm, 3 mm	Delamination test
Without origami	-	-	

**Table 4:** Specimen setup.

Specimen	Structure Thickness (mm)	Dimensions (mm) (Length/Width)	Structure Species
With origami capsule	5 mm	193 L/30 W	
Without origami capsule	5 mm	193 L/30 W	With holes and pillars, see Figure 4b

**Table 5:** Various values of coefficients for simple TPE “cross” origami capsules of different thicknesses.

Thickness (mm)	*T*_00_	*T*_10_	*T*_01_	*T*_20_	*T*_11_	R-Square
1 mm	0.2492	−137	2.373	2086	−16.41	0.9991
2 mm	0.7806	389.9	0.3744	513.1	−18.49	0.994
3 mm	0.6838	86.07	1.134	329.1	−8.55	0.9965

**Table 6:** Experimental and theoretical value of strain energy release of the structure (a) with origami and (b) without origami.

	With Origami (5 mm)	Without Origami (5 mm)
The experimental value of strain energy release	ϵ=3.831	ϵ=2.555
Theoretical model value of strain energy release	ϵ=3.238	ϵ=2.072
Percent deviation in strain energy release	∆ϵ = 0.183 (18.31%)	∆ϵ = 0.233 (23.3%)

## Data Availability

The data presented in this study are available on request from the corresponding author.

## References

[1] Zhang Z., Demir K.G., Gu G.X. (2019). Developments in 4D-Printing: A Review on Current Smart Materials, Technologies, and Applications. Int. J. Smart Nano Mater..

[2] He F., Thakur V.K., Khan M. (2021). Evolution and New Horizons in Modeling Crack Mechanics of 3D Printing Polymeric Structures. Mater. Today Chem..

[3] He F., Khan M. (2021). Effects of Printing Parameters on the Fatigue Behaviour of 3d-Printed Abs under Dynamic Thermo-Mechanical Loads. Polymers.

[4] Chu H., Yang W., Sun L., Cai S., Yang R., Liang W., Yu H., Liu L. (2020). 4D Printing: A Review on Recent Progresses. Micromachines.

[5] Yang P., Zhu F., Zhang Z., Cheng Y., Wang Z., Li Y. (2021). Stimuli-Responsive Polydopamine-Based Smart Materials. Chem. Soc. Rev..

[6] Khan N.I., Halder S., Gunjan S.B., Prasad T. (2018). A Review on Diels-Alder Based Self-Healing Polymer Composites. IOP Conf. Ser. Mater. Sci. Eng..

[7] Willocq B., Odent J., Dubois P., Raquez J.M. (2020). Advances in Intrinsic Self-Healing Polyurethanes and Related Composites. RSC Adv..

[8] Li X., Yu R., He Y., Zhang Y., Yang X., Zhao X., Huang W. (2019). Self-Healing Polyurethane Elastomers Based on a Disulfide Bond by Digital Light Processing 3D Printing. ACS Macro Lett..

[9] Zentner C.A., Anson F., Thayumanavan S., Swager T.M. (2019). Dynamic Imine Chemistry at Complex Double Emulsion Interfaces. J. Am. Chem. Soc..

[10] Zhang Z.P., Rong M.Z., Zhang M.Q. (2018). Mechanically Robust, Self-Healable, and Highly Stretchable “Living” Crosslinked Polyurethane Based on a Reversible C–C Bond. Adv. Funct. Mater..

[11] Almutairi M.D., Aria A.I., Thakur V.K., Khan M.A. (2020). Self-Healing Mechanisms for 3D-Printed Polymeric Structures: From Lab to Reality. Polymers.

[12] Anand L.D.V., Hepsiba D., Palaniappan S., Sumathy B., Vijayakumar P., Rani S.S. (2021). Automatic Strain Sensing Measurement on Steel Beam Using Strain Gauge. Mater. Today Proc..

[13] Alshammari Y.L.A., He F., Khan M.A. (2021). Modelling and Investigation of Crack Growth for 3d-Printed Acrylonitrile Butadiene Styrene (Abs) with Various Printing Parameters and Ambient Temperatures. Polymers.

[14] Meulman E., Renart J., Carreras L., Zurbitu J. (2023). A Methodology for the Experimental Characterization of Energy Release Rate-Controlled Creep Crack Growth under Mode I Loading. Eng. Fract. Mech..

[15] Santos P., Silva P. (2023). Effect of Carbon Nanofibers on the Strain Rate and Interlaminar Shear Strength of Carbon/Epoxy Composites. Materials.

[16] Wen N., Song T., Ji Z., Jiang D., Wu Z., Wang Y., Guo Z. (2021). Recent Advancements in Self-Healing Materials: Mechanicals, Performances and Features. React. Funct. Polym..

[17] Speck O., Speck T. (2019). An Overview of Bioinspired and Biomimetic Self-Repairing Materials. Biomimetics.

[18] Xiong C., Wang T., Zhao Z., Ni Y. (2023). Recent Progress in the Development of Smart Supercapacitors. SmartMat.

[19] Irzhak V.I., Uflyand I.E., Dzhardimalieva G.I. (2022). Self-Healing of Polymers and Polymer Composites. Polymers.

[20] Grammatikopoulos A., Banks J., Temarel P. Experimental Dynamic Properties of ABS Cellular Beams Produced Using Additive Manufacturing. Proceedings of the 18th European Conference on Composite Materials.

[21] Mondal S., Ghuku S., Saha K.N. (2018). Effect of Clamping Torque on Large Deflection Static and Dynamic Response of a Cantilever Beam: An Experimental Study. Int. J. Eng. Technol..

[22] Brown E.N., Sottos N.R., White S.R. (2002). Fracture Testing of a Self-Healing Polymer Composite. Exp. Mech..

[23] Siddique A., Abid S., Shafiq F., Nawab Y., Wang H., Shi B., Saleemi S., Sun B. (2021). Mode I Fracture Toughness of Fiber-Reinforced Polymer Composites: A Review. J. Ind. Text..

[24] Pati S., Singh B.P., Dhakate S.R., Ponnamma D., Sadasivuni K.K., Cabibihan J.J., Al-Maadeed M.A.-A. (2017). Self-Healing Polymer Composites Based on Graphene and Carbon Nanotubes.

[25] Hu Z., Zhang D., Lu F., Yuan W., Xu X., Zhang Q., Liu H., Shao Q., Guo Z., Huang Y. (2018). Multistimuli-Responsive Intrinsic Self-Healing Epoxy Resin Constructed by Host-Guest Interactions. Am. Chem. Soc..

[26] Peraza Hernandez E.A., Hartl D.J., Lagoudas D.C. (2019). Structural Mechanics and Design of Active Origami Structures. Active Origami.

[27] He F., Ning H., Khan M. (2023). Effect of 3D Printing Process Parameters on Damping Characteristic of Cantilever Beams Fabricated Using Material Extrusion. Polymers.

[28] Li Y., You Z. (2019). Open-Section Origami Beams for Energy Absorption. Int. J. Mech. Sci..

[29] Mashkoor F., Lee S.J., Yi H., Noh S.M., Jeong C. (2022). Self-Healing Materials for Electronics Applications. Int. J. Mol. Sci..

[30] Zhang W., Zheng Q., Ashour A., Han B. (2020). Self-Healing Cement Concrete Composites for Resilient Infrastructures: A Review. Compos. B Eng..

[31] Nodehi M., Ozbakkaloglu T., Gholampour A. (2022). A Systematic Review of Bacteria-Based Self-Healing Concrete: Biomineralization, Mechanical, and Durability Properties. J. Build. Eng..

[32] Maddalena R., Bonanno L., Balzano B., Tuinea-Bobe C., Sweeney J., Mihai I. (2020). A Crack Closure System for Cementitious Composite Materials Using Knotted Shape Memory Polymer (k-SMP) Fibres. Cem. Concr. Compos..

[33] Zai B.A., Khan M.A., Khan S.Z., Asif M., Khan K.A., Saquib A.N., Mansoor A., Shahzad M., Mujtaba A. (2020). Prediction of Crack Depth and Fatigue Life of an Acrylonitrile Butadiene Styrene Cantilever Beam Using Dynamic Response. J. Test Eval..

[34] Jagadeesh P., Puttegowda M., Rangappa S.M., Alexey K., Gorbatyuk S., Khan A., Doddamani M., Siengchin S. (2022). A Comprehensive Review on 3D Printing Advancements in Polymer Composites: Technologies, Materials, and Applications.

[35] Dhaliwal G.S., Dundar M.A. (2020). Four Point Flexural Response of Acrylonitrile–Butadiene–Styrene. J. Compos. Sci..

[36] Lee T.U., You Z., Gattas J.M. (2018). Elastica Surface Generation of Curved-Crease Origami. Int. J. Solids Struct..

[37] Damas S.M., Turner C.J. The Material Testing of Nanoparticle Doped 3D Printed ABS Strain Gages for Resistance and Stiffness. Proceedings of the International Design Engineering Technical Conferences and Computers and Information in Engineering Conference IDETC/CIE2020.

[38] Ritzen L., Montano V., Garcia S.J. (2021). 3d Printing of a Self-Healing Thermoplastic Polyurethane through Fdm: From Polymer Slab to Mechanical Assessment. Polymers.

[39] UL Prospector Generic Families of Plastic. https://www.ulprospector.com/plastics/en/generics.

[40] Srivastava V.K. (2017). A Review on Advances in Rapid Prototype 3D Printing of Multi-Functional Applications. Sci. Technol..

[41] Almutairi M.D., Khan M.A. (2022). Experimental Investigation of Polymeric Beam Under Elastic and Plastic Loads. Int. J. Appl. Phys. Sci..

[42] Almutairi M.D., Alnahdi S.S., Khan M.A. (2022). Strain Release Behaviour during Crack Growth of a Polymeric Beam under Elastic Loads for Self-Healing. Polymers.

[43] Blaiszik B.J., Kramer S.L.B., Olugebefola S.C., Moore J.S., Sottos N.R., White S.R. (2010). Self-Healing Polymers and Polymer Composites. Annu. Rev. Ofmater. Res..

[44] Drake D.A., Sullivan R.W., Lovejoy A.E., Clay S.B., Jegley D.C. (2021). Influence of Stitching on the Out-of-Plane Behavior of Composite Materials—A Mechanistic Review. J. Compos. Mater..

[45] Kessler M.R., Sottos N.R., White S.R. (2003). Self-Healing Structural Composite Materials. Compos. Part A Appl. Sci. Manuf..

